# Health Systems Readiness to Manage the Hypertension Epidemic in Primary Health Care Facilities in the Western Cape, South Africa: A Study Protocol

**DOI:** 10.2196/resprot.5381

**Published:** 2016-02-29

**Authors:** Rodrigue Innocent Deuboué Tchialeu, Sanni Yaya, Ronald Labonté

**Affiliations:** ^1^ Population Health Program Faculty of Health Sciences University of Ottawa Ottawa, ON Canada; ^2^ School of International Development and Global Studies Faculty of Social Sciences University of Ottawa Ottawa, ON Canada; ^3^ Faculty of Medicine University of Ottawa Ottawa, ON Canada

**Keywords:** Hypertension, Manage, Health System, Epidemics, Scaling-up Interventions, Requirements, Supply Chain, Antihypertensive agents, Control, Delivery of Health Care

## Abstract

**Background:**

Developing countries are undergoing a process of epidemiological transition from infectious to noncommunicable diseases, described by the United Nations Secretary General Ban Ki-Moon as ‘‘a public health emergency in slow motion.” One of the most prevalent in sub-Saharan Africa is hypertension, which is a complex chronic condition often referred to as a “silent killer” and key contributor to the development of cardiovascular and cerebrovascular diseases. Hypertensive patients in this setting are estimated to increase from 74.7 million in 2008 to 125.5 million in 2025, a 68% increase. However, there is an important gap between emerging high-level policies and recommendations, and the near-absence of practical guidance and experience delivering long-term medical care for noncommunicable diseases within resource-limited health systems.

**Objective:**

To address this gap, our study will consist of field investigations to determine the minimum health systems requirements to ensure successful delivery of antihypertensive medications when scaling-up interventions to control the hypertension epidemic.

**Methods:**

A cross-sectional analytic study will be conducted in the Western Cape using a mixed-method approach with two semistructured interview guides. The first will be for health professionals involved in the care of hypertensive patients within at least 6 community health centers (3 urban and 3 rural) to understand the challenges associated with their care. The second will be to map and assess the current supply chain management system of antihypertensive medications by interviewing key informants at different levels of the processes. Finally, modeling and simulation tools will be used to understand how to estimate minimum numbers of health workers required at each supply chain interval to ensure successful delivery of medications when scaling-up interventions.

**Results:**

Funding for the study was secured through a Doctoral Research Award in October 2014 from the International Development Research Centre (IDRC). The study is currently in the data analysis phase and results are expected during the first half of 2016.

**Conclusions:**

This investigation will highlight the detailed processes in place for the care of hypertensive patients in primary health care facilities, and thus also identify the challenges. It will also describe the drug supply chain management systems in place and identify their strengths and weaknesses. The findings, along with the estimates from modeling and simulation, will inform the health system minimum requirements to scale-up interventions to manage and control the hypertension epidemic in the Western Cape province of South Africa.

## Introduction

### Background and Statement of the Problem

Noncommunicable diseases (NCDs) ‘‘have no borders or boundaries—they are the world’s number one killer and devastate the bottom billion and G20 countries alike’’ [1]. Developing countries are undergoing a process of epidemiological transition from infectious to noncommunicable diseases. The launch of the World Health Organization Action Plan on NCDs, the UN General Assembly Resolution on the Prevention and Control of NCDs, and the Global Alliance for Chronic Diseases (GACD) provide strong evidence that increasing attention is being paid to the impact of noncommunicable diseases on health and development. The United Nations Secretary General Ban Ki-Moon described NCDs as ‘‘a public health emergency in slow motion” [2]. One of the most prevalent NCDs in sub-Saharan Africa (SSA) is hypertension. Hypertension is a complex chronic condition, often referred to as the “silent killer” and a key contributor to the development of cardiovascular and cerebrovascular diseases. Hypertension is often either not diagnosed or untreated, and thus represents a serious unrecognized epidemic in Africa. In 2008, the prevalence of hypertension in SSA was estimated at 16.2%, and it was predicted that in 2025 the figure would rise to 17.4% (95% CI 15.4%-22.6%). As a result, the number of hypertensive patients will increase from 74.7 million in 2008 to 125.5 million in 2025—a 68% increase [3]. A recent systematic review including a meta-analysis predicted the prevalence of hypertension in SSA at mean participant ages of 30, 40, 50, and 60 years to be 16%, 26%, 35%, and 44%, respectively, with a pooled prevalence of 30%. Of those with hypertension, only between 7% and 56% (pooled prevalence 27%; 95% CI 23%-31%) were aware of their hypertensive status before the surveys. Overall, 18% (95% CI 14%-22%) of individuals with hypertension were receiving treatment across the studies, and only 7% (95% CI 5%-8%) had controlled blood pressure. This important and timely study concluded that high prevalence of hypertension, low percentage of awareness, treatment, and control in SSA call for implementation of timely and appropriate strategies for diagnosis, control, and prevention [4].

South Africa, however, has one of the highest rates of hypertension in SSA, where the condition has been well researched compared to other countries in the region. The prevalence of hypertension in the South African population is as high as 44% in adult males living in rural areas. In addition, older adults and males are more affected by hypertension in South Africa [5]. However, unlike other countries in the region, the prevalence has been reported as comparable between rural and urban populations [6], which is attributed to an increase in obesity caused by widespread adoption of Westernized diets throughout the country. The South African National Health and Nutrition Examination Survey (SANHANES-1), released in 2013, revealed that among participants who had undergone a clinical examination and had their blood pressure measured, an increasing percentage of the population had systolic blood pressures that were high (≥ 140 mmHg) in the group 15-25 years of age (5.3%) compared to those 65 years of age and older (63.7%). Half (50.5%) of participants 55-64 years of age had a high systolic blood pressure [7].

Diagnosis, treatment, and control of blood pressure are a major population health problem. Screening campaigns are largely nonexistent because of the generally poor health care infrastructure and low number of health care workers. Most systems cannot afford the additional costs associated with operating a cardiovascular screening program in an already resource-limited environment. Thus, there is an important gap between emerging high-level policies and recommendations, and the near-absence of practical guidance and experience delivering long-term medical care for patients with NCDs within resource-limited health systems.

### Purpose of the Study and Significance

To address this gap, our research will consist of field investigations in South Africa to study the implementation and scale-up of diagnosis and drug treatment systems for hypertension. In the context of scaling up access to antihypertensive medications in resource- and infrastructure-limited health care systems, this study will specifically examine the development of an effective drug supply management system and study how to determine the minimum numbers of health care workers, and at what level of training, required at each supply chain interval to ensure adequate care of patients. Our overall goal is to determine the minimum health care system requirements to ensure successful delivery of antihypertensive medications when scaling-up interventions to control the hypertension epidemic in South Africa. The rationale for choosing South Africa for these investigations is that, to our knowledge, it has the most clearly documented hypertension epidemic in SSA, and South African medical care frequently guides other African countries.

### Theoretical/Conceptual Framework

Our focus is on the supply chain as a key element of system responsiveness to hypertension, because to address such an epidemic and reach the maximum number of people, effective and well-structured procedures need to be in place. In addition, a well-functioning supply chain system ensures cost-effectiveness of interventions. In order to be sustained and to expand, a supply chain needs to be robust, agile, and flexible. Systems need to be in place to account for potential reductions in annual funding, increases in drug costs, decreases in drug quality, and loss of drug products. Should any of these situations arise, the management system needs to be designed in such a way that available drugs are equitably distributed to those most in need. Policy makers, program and supply chain managers are under intense pressure to improve health care supply chains to ensure an uninterrupted flow of drugs and related supplies at various service delivery points. They are also under pressure to produce greater levels of responsiveness, shorter lead times and cycle times for delivery, and positioning of high-quality drug inventory and related commodities to meet the health care needs of millions of people. All these parameters make any scale-up process fairly complex.

**Figure 1:**
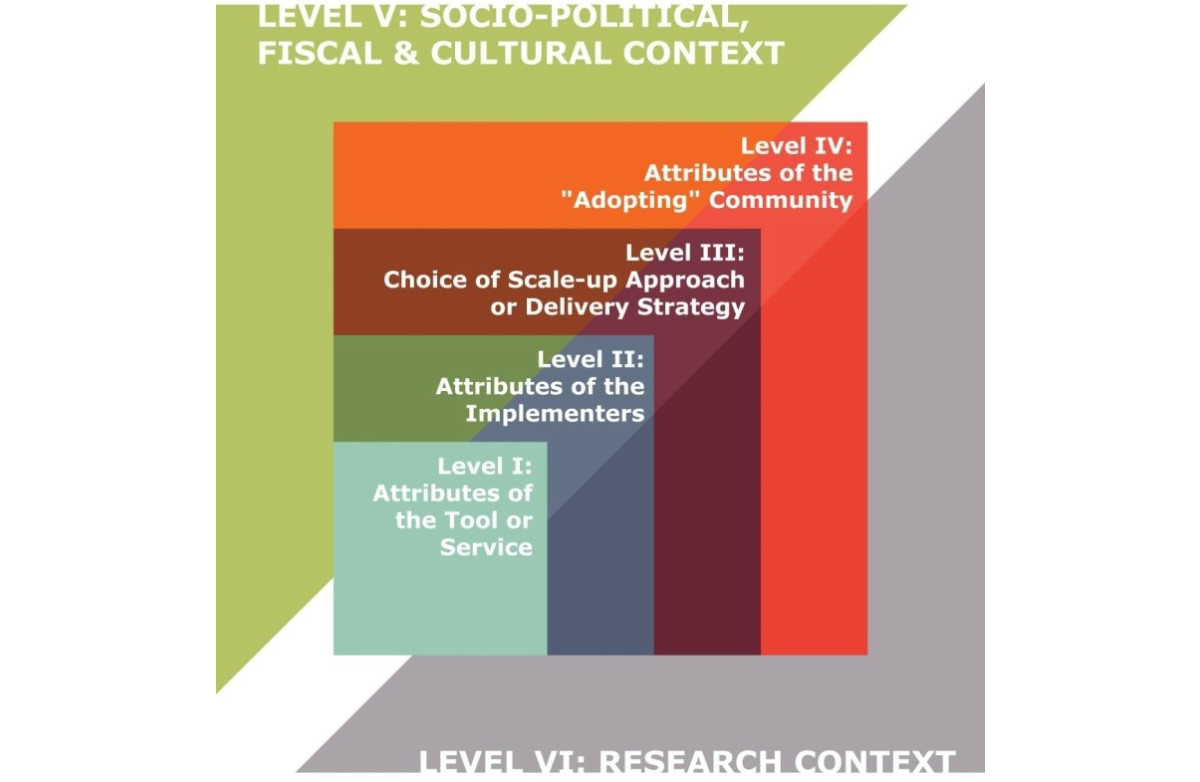
Framework for categorizing the study results—The Yamey Framework.

Our proposal aims to address these key aspects of the scale-up of an intervention by examining health system requirements to ensure successful delivery of medications to control the hypertension epidemic in South Africa. In order to serve this purpose, we have chosen to use the framework proposed by Gavin Yamey in 2012, hereafter referred to as the “Yamey Framework,” illustrated in Figure 1 [8]. We chose this framework because it draws on insights from interviews with scale-up “leaders,” many of whom have led national or global health implementation programs, and incorporates themes emerging from relevant recent literature. This framework takes full advantage of the “learning by doing” concept in ways that engage key stakeholders, uses data to address constraints, and incorporates results from pilot projects. Such approaches are very relevant for tackling issues that arise when scaling-up interventions.

The Yamey Framework identifies a range of reported success factors, which are organized into 6 categories, representing different components of the scaling-up process. Inspired by the work of Bergh, van Rooyen, and Pattinson (2008) and Simmons and Shiffman (2007), these categories are the following: attributes of the specific tool or service being scaled-up, attributes of the implementers, chosen delivery strategy, attributes of the “adopting” community, the socio-political context, and the research context [9,10].

#### Attributes of the Tool or Service Being Scaled-Up

This framework identifies the importance of simplicity of the intervention and scientifically robust technical policies. Keeping the intervention simple is widely considered to be an important predictor of success. In addition, technical experts who have managed large-scale implementation argue that ensuring technical policies are scientifically robust before going to scale is crucial for success [11].

#### Attributes of the Implementers

Strong leadership and governance, engaging local implementers and other stakeholders, and using both state and non-state actors as implementers are the key attributes recommended by the framework. These features will be particularly relevant for us in selecting our key informants for the mapping and assessment of the current supply chain management system of antihypertensive medications in South Africa, particularly in the Western Cape region.

#### Chosen Delivery Strategy

In this category, the framework recommends applying diffusion and social network theories, applying cascade and phased approaches to scale-up, tailoring scale-up to the local situation, decentralizing delivery, and adopting an integrated approach to scale-up. Important lessons from limitations of purely vertical or horizontal interventions prompt more emphasis on integrated approaches.

#### Attributes of the “Adopting” Community

An engaged, “activated” community is needed. The active participation of the community in planning, implementing, and monitoring interventions is widely cited as a crucial factor in successful scale-up [12]. This is important to avoid blind transfer of solutions from developed to developing countries without integrating local norms.

#### Socio-Political Context

Political will, national policies, and country leadership are key points. Clear and easy-to-implement policies should be in place to ensure successful scaling-up of the intervention. This aligns well with the concept of a “policy window,” which is very important in population health research for the success of interventions, that is, the timing of proposed interventions should be compatible with the political agenda of policy makers. Moreover, health policy research has focused largely on the content of policy, neglecting actors, context, and processes [13]. These four elements should be taken into consideration, and in particular, how these elements interact to shape policy-making. This component of the framework articulates the notion of sustainability, a very important principle of effective population health interventions that we plan to integrate into our research.

#### Research Context

Incorporating research into implementation (“learning and doing”) is the last feature of the framework. We mentioned this earlier as one the main reasons why we chose this framework. Some have argued that successful scale-up “requires the systematic use of evidence to guide the process and incorporate new learning” [10].

### Research objectives

Our overall objective in this study is to determine health care system requirements to ensure successful delivery of antihypertensive medications when scaling-up interventions to manage and control the hypertension epidemic in South Africa and the Western Cape Province in particular. No a priori hypotheses have been formulated. Instead, this program of research will use a combination of primary, administrative, and survey data to undertake the necessary analyses to meet the following three specific objectives:

1. To understand the challenges of the health care system in the care of hypertensive patients;

2. To map and assess the current supply chain management system of antihypertensive medications in the Western Cape province of South Africa;

3. Using modeling and simulation tools, to understand how to determine the minimum numbers of health care workers required at each supply chain interval to ensure successful delivery of medications when scaling-up interventions.

## Methods

The overarching research question of this thesis proposal is to determine the health care system requirements to ensure successful delivery of antihypertensive medications in the developing country of South Africa when scaling-up interventions to control the epidemic of hypertension. System requirements include materials and human resources linked by processes and policies. This research question will be addressed using a variety of research methods appropriate to each subresearch question presented here, which are informed by the theoretical framework.

### Study Design

**Figure 2:**
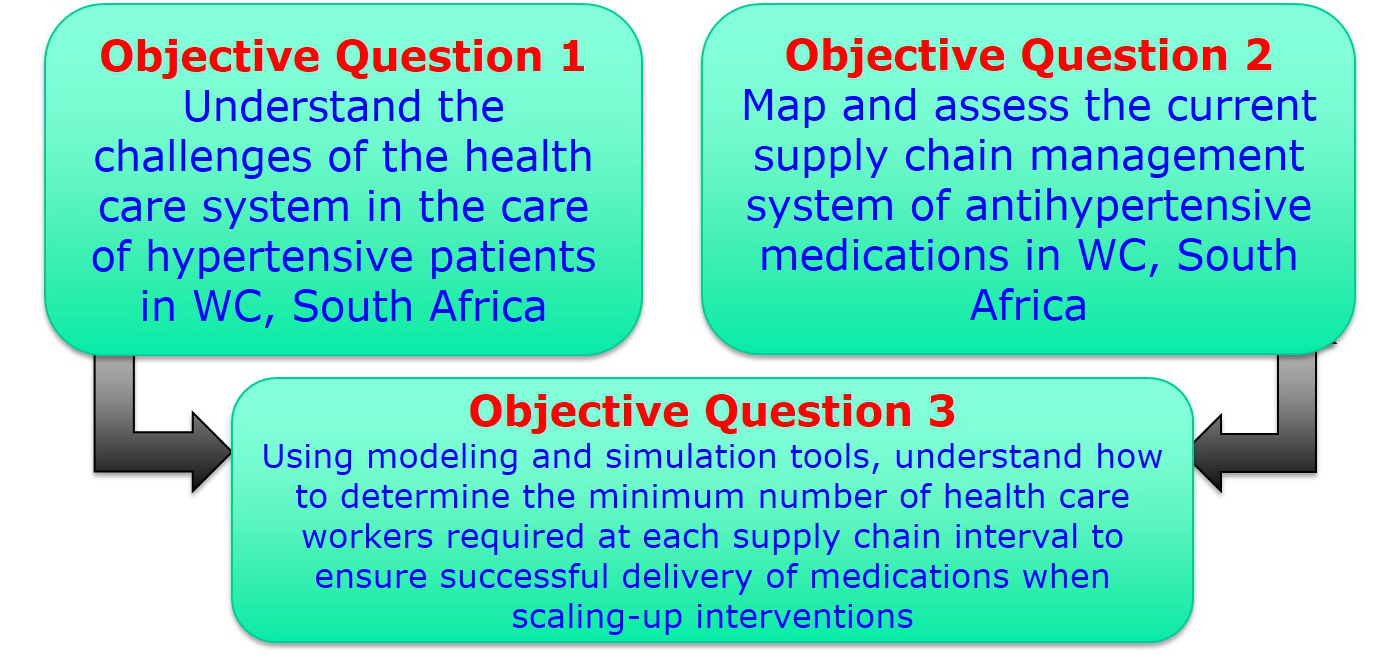
Linked study objectives.

This combined qualitative and quantitative study will be designed around 3 specific objectives (see Figure 2).

At a very broad level, an antihypertensive medication supply chain will include processes to quantify the drugs needed, procure the drugs, store and control the inventory, distribute the drugs to clinics, and ensure an adequate level of quality assurance. We will conduct field work and have access to secondary data through the South African Medical Research Council (SA MRC). We chose the Western Cape region of South Africa for our field investigations mostly because of the facilitated access through the SA MRC. We recognize that the epidemiology of hypertension may vary from one region to another; however, our research mainly focuses on procedures and operations within the health care system that are comparable between regions. Results obtained in the Western Cape region certainly won’t be generalizable throughout the country; however, they could serve as a reference for similar studies in other regions and methods and models could also be replicated.

First, our study will assess the challenges of the health care system, particularly in the Western Cape region, in the care of hypertensive patients. An assessment of standard procedures in a number of health care facilities will identify key processes and individuals’ responsibilities in diagnosis and treatment provision. We will combine secondary and primary data to perform this assessment. Primary data will be collected through key informant interviews of medical personnel in the form of semi-structured questionnaires. Data collected will help to describe how patients are processed at the health center, from arrival through health assessments and medication collection to departure, with details on individual processes and health care facility personnel involved. Individual processes for each type of health care facility involved will focus on elaborating current tasks with duration estimation, and identifying potential tasks that could be added or removed to inform task-shifting options. Key informants will be facility managers and health care providers (nurses, pharmacists, doctors, allied healthcare providers) working in health centers of the Western Cape region in South Africa. We will select at least 6 public health care facilities equally distributed between the Cape Town metro (urban) and rural area. The main inclusion criterion for the selection of a health care facility for our study is the implementation of at least a weekly dedicated hypertension or chronic NCD treatment clinic. Secondary data to complement our field investigation, such as administrative hospital data and pharmacy inventory data will be gathered at the health care facilities. Overall, through primary and secondary data analysis, we expect to obtain information on levels of staffing, hypertension screening practice in the health center (routine or opportunistic), volume of patients visits over a certain period such as the last 12 months (% with hypertension), average time allocated to patient per consultation, presence of task-shifting mechanisms, level of adherence to treatment, availability of drugs at the site and cost to hypertensive patients, frequency of drug shortages, presence of an equitable distribution policy during drug shortages, blood pressure measurement equipment and servicing frequency, and nurse confidence in successfully delivering tasks outside of the traditional scope of attribution in regard to chronic disease management. Data will be analyzed with consideration of sex and gender to determine if there are unique sex and gender differences in the control of hypertension. Data collected in public health centers will be analyzed qualitatively and quantitatively to understand the current state of practice and highlight strengths and challenges of the health care system in regards to hypertension diagnosis and treatment.

**Figure 3:**
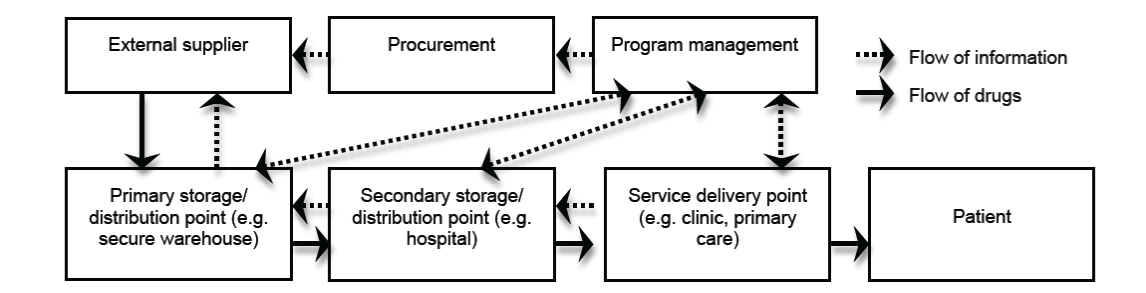
Supply chain management framework (Persson, 1995).

Second, the study will investigate the current state of the drug supply chains for antihypertensive medications in South Africa, particularly in the Western Cape region, using an exploratory design. This will be done through mapping of the current public drug supply chains for antihypertensive medications, which will uncover gaps, weaknesses, and strengths of the current supply chain. Practically, we will describe the supply chain by following the flow of medications backward from the service delivery point or health center, to the program management unit where drug selection and quantification take place. This investigation will require interviewing key informants at each step to determine operational procedures, stakeholders, and next steps. Semi-structured interviews will be administered with both closed and a few open-ended questions. Other individuals who will be identified during interviews will also be contacted for their input. In addition, secondary data available at each step will be collected and analyzed to estimate trends, frequencies, uncertainties or fluctuations, lead times, stock shortages, volumes, and potential needs when scaling-up. As we build on existing infrastructure, scaling-up services in this project will involve doing more than what is currently achieved by the health care system in terms of coverage, quality of services, and cost effectiveness of processes. This process will help to identify factors that either enhance or hinder accessibility to antihypertensive drugs for South African population sectors vulnerable to hypertension epidemics. In addition, it will develop a comprehensive supply chain map that will reveal the labyrinth of the South African logistics infrastructure, distribution channels, government regulations, and business customs. The identification of potential leverage points will also inform our strategy in scaling-up interventions to control the epidemic of hypertension. A basic model for logistics process redesign will serve as the basis for describing and analyzing logistics processes involved in the flow of information and goods of the public drug supply chain (see Figure 3) [14].

At the management level, we will select key informants from the provincial department of health (DOH) and use available secondary data to identify procedures in place to gather information estimating how much of each drug is needed. For the procurement level, which pertains to selecting suppliers, placing and monitoring orders, checking delivery quantities and quality, and paying suppliers, key informants at the DOH, the unit in charge of drug supply will be interviewed, combined with available data to identify lead times, ordering frequencies, and demand patterns. Data from this level will also help identify major suppliers and lead times. The distribution component will be investigated by interviewing managers at these levels to gather information on order reception, storage, stock control, transportation, and record keeping for monitoring and control, which will be analyzed to determine shortage frequencies, inventory management practices, stock fluctuations, and uncertainties. The service delivery points will provide data on prescription, dispensing and use of drugs, and patients’ compliance with prescriptions. We will complement our investigation by using various data sources from the South African National Health and Nutrition Survey (SANHANES), Hypertension Society of Southern Africa, trade, government reports, manufacturers, and customs to perform our mapping. We expect to identify and evaluate the key elements and processes of the current supply chain management system of antihypertensive medications in South Africa, particularly in the Western Cape region. Special attention will be given to principal public actors (government departments, various levels), volumes of supplies per year, cost of supplies per year, majors suppliers, frequencies of orders, transportation actors and processes, shortage management strategies, variation of demands level, storage conditions and processes, inventory management, quality assurance processes, staffing levels and numbers at each interval, volume level at each storage level, monitoring and record keeping of drug availability, and utilization patterns. We will focus mainly on public sector primary healthcare clinics known as Community Health Centers (CHCs) for this study, as they provide a network of accessible and free primary care for acute and chronic illnesses. We believe that they are fairly representative of clinical practices for the vast majority of hypertensive patients and the SA MRC, which is our host for the study, has agreed to facilitate access to public sector primary health care clinics in the Western Cape region, with which they already have extensive collaboration. This will help uncover the gaps, weaknesses, and strengths of the current public drug supply chain management system and inform the implementation of the model.

Finally, we will explore and use modeling and simulation tools to determine how to estimate the minimum number of health workers required at service delivery points to ensure successful delivery of sufficient medications when scaling-up interventions. In the event that authorities would like to put programs in place to increase diagnosis and treatment of hypertensive patients (ie, scaling-up interventions), modeling and simulation will help to determine what would be needed of the health care system to tackle such a volume of patients. Our study is not actually implementing any scaling-up activity. Data analysis of the earlier study component on how hypertensive patients are processed at health centers, with details on individual processes including task descriptions with time duration estimations, will provide parameters for our modeling and simulations. Tasks and time duration estimations per task will be computed in our modeling to estimate the minimum number of each type of health care worker needed when scaling-up interventions. Quantification of drug needs at service delivery points, which will also be included in the model, will be performed using the World Health Organization’s (WHO) “Morbidity Method” [15]. In the standard version of the “Morbidity Method,” the total quantity of drugs required for a health problem is given by the quantity of drugs given as a standard treatment for the problem, multiplied by the number of treatment episodes of that problem. Modeling and simulations will be executed using known hypertension prevalence estimates as inputs. Quantifying the requirement for medication is an essential step in the overall process of medication procurement with the aim of ensuring access. Quantification seeks to answer the question “How much of each medication is needed?” It is the process of determining the quantity of each selected medicinal product required to meet the needs of a specified location (clinic, hospital, region, or country) or program (eg, TB program) for a specified period of time. In addition to being a step in the procurement process, quantification of requirements also provides vital information and a basis for managing the distribution of medication [16].

### Participants

In this study, our stakeholders are of various types and levels but inform the analysis in a concurrent manner. We will interview only policy makers, administrative staff involved with the drug supply chain, and health care personnel within a few public health centers. No patient will be interviewed as the focus is on processes followed by providers within the health care systems. There will be no translation issues as we expect to use only English when speaking with professional staff. As a general rule, only individuals who have been in their positions for at least 3 months will be included in the study. Each participant will sign a consent form to participate after a verbal briefing about the purpose and structure of the study.

With regard to the mapping of the current public drug supply chain for antihypertensive medications in South Africa, participants will be identified at each stage of the Supply chain management framework. As described earlier in the Study Design section, key informants will be interviewed in project management, procurement, storage, distribution, and service delivery points. This includes but is not limited to key individuals in the Western Cape DOH, pharmacists, and those involved in the procurement, storage, and distribution of medication. Any other organizations or key individuals identified during interviews that may provide useful or additional insight will be asked to participate as well.

With respect to studying how patients are processed at health centers from arrival to departure, with details on individual processes and health care facility personnel involved, participants will be facility managers and health care providers such as nurses, pharmacists, doctors, and allied healthcare providers. This will be an iterative process, such that some key stakeholders may be identified later in the process.

### Data Collection Techniques

In this study, the techniques to collect data will be interviews, review of administrative data, and document review. There will be 2 semi-structured questionnaires: one for managers and policymakers in ministries and agencies and the other for medical personnel involved with the hypertension program in selected public hospitals. We will conduct face-to-face interviews; however, in cases for which arranging a meeting will be difficult, telephone interviews will be used as an alternative. We chose to have only open-ended questions in a semi-structured interview format because this gives respondents flexibility to organize and articulate fully their responses [17]. All interviews will be held at the offices of the respondents or at a place and time of their own convenience. At the beginning of every interview, I (the first author) will introduce myself, the purpose of my research, who is funding my research, how I will disseminate the findings of my research, and ask for permission to tape the interview. At the end of the interview, I will ask respondents if they have any comments or questions regarding my research, and also about their perceptions of the current hypertensive drugs supply chain with an emphasis on potential barriers and enablers. This will be helpful in knowing whether I have overlooked critical questions. I will also ask for follow-up interviews to clarify information I do not understand.

Data to inform mapping will be obtained through published survey data and reports, a review of the literature, and interviews with managers and policy makers in ministries and agencies. We will be guided by the elements of the supply chain management framework in the selection of the stakeholders to interview and we also anticipate a “snowball” effect as we progress with our data collection. This is possible because the actual local reality of the supply chain in the country may not completely match our framework. Here, qualitative data will be collected to understand the mechanisms underlying the flow of hypertensive drugs to and within the country, and also to clearly identify all relevant actors. Quantitative data will provide information on volumes, costs and actors as well. A combination of field research, literature review, regulations review, and track record review will be put in place.

Data for the development and testing of the model will be obtained from interviews of medical personnel involved with the hypertension program in selected public hospitals. Secondary data will be obtained from document reviews and administrative databases through the South African Medical Research council regarding hypertension morbidity, demographic health surveys, training records of health care professionals of various levels, numbers of health care workers per level and region, antihypertensive drug consumption, and drug import data. Data from hospital records on screening, follow-up visits, availability of health care personnel, wait times, and daily average numbers of patients received will also be extracted.

All primary data will be obtained through semi-structured questionnaires that will be recorded and transcribed before analysis. Consent will be obtained for recording the interviews and no personal information will be recorded. We plan on obtaining input from knowledgeable individuals of the Western Cape Department of Health to finalize our questionnaires, which can only be done once approval for the study is obtained from the Ethics Committees.

Secondary data will be mostly administrative data and document review. Administrative data will be extracted from accessible surveys such as the South African National Health and Nutrition Examination Survey (SAHANES) and records from health institutions and agencies where we will interview study participants. These data will inform us on the current processes and descriptive statistics will be computed. We will also gather information through document review for the same purposes. Secondary data in this study project will mostly complement primary data gathered through semi-structured interviews. As we approach key informants and health professionals, we will request access to available administrative data and documents. Secondary data will also help us to adapt our semi-structured questionnaire to ask more effective questions according to our research objectives.

### Data Analysis

Transcripts will be sent to respondents for their validation, if requested, before data analysis begins. Interview data collected from the field will be coded into 2 basic types: manifest and latent. Manifest coding refers to direct responses to particular questions by respondents. Latent coding considers responses that were not explicitly called for by the question [17]. Themes will be developed out of the interview transcripts and content analysis will also be used to analyze documents. A structured approach guided by the semi-structured interview will be used to determine salient themes emerging from the interviews.

To understand the current state of the drug supply chains for antihypertensive medications in South Africa, data collected through interviews of managers and policy makers in the ministries and agencies will be analyzed to identify social actors, various connections, and processes involved in the drug supply chains for antihypertensive medications in South Africa. These elements will be inputted into the CmapTools software [18] to produce a map organizing and representing knowledge extracted from the data about the current state of the drug supply chains for antihypertensive medications in South Africa. This comprehensive supply chain map will reveal the labyrinth of the South African logistics infrastructure, distribution channels, government regulations, business customs, gaps, weaknesses, and strengths related to the antihypertensive drug supply chain.

Data collected for the development of the model through interviews of medical personnel involved with the hypertension program in selected public hospitals will be analyzed using the qualitative software Atlas ti. We will build a model to determine the necessary drug supply and human resource requirements for the supply chain. Models have proven to be effective forecasting tools for drug supply chains, even in low-resource settings. The model will start at the point of quantification and procurement, and will include the subsequent processes of primary and secondary storage, transportation, and service delivery. The model will also include mechanisms to account for changes in drug demand and will assess different scenarios of procurement, information exchange, and shipment processes [19]. Costs will be integrated at each level of the model to ensure that I (the first author) can create scenarios that are not only cost-efficient, but effective in ensuring drugs reach the patient in a timely manner. The Supply Chain Intelligence software package available through SAS will be used to execute the model [20]. I expect that this study will determine the minimum number of health care workers, and at what level of training, are required according to population density and size.

We would like to be able to return to our key informants, stakeholders, and participants with our findings and recommendations, not only to disseminate and promote knowledge translation, but also to obtain their feedback and comments that will help to fine-tune, validate, and optimize the mathematical model used.

## Results

Funding for the study was secured through a Doctoral Research Award in October 2014 from the International Development Research Centre (IDRC). Approximately 12 months were spent in the field organizing data collection activities, and requesting and obtaining local ethics approval and authorization to access the specific health care facilities and proceed with data collection. Health care providers involved in the care of hypertensive patients were interviewed in 9 primary health care centers (4 rural and 5 urban, around the Western Cape Province) for a total of 54 participants in this part of the study. In addition, 18 key informants at various levels of the pharmaceutical supply chain system in the Western Cape Province were interviewed. Over 55 hours of recordings were collected by myself and then transcribed through a professional third party organization in Cape Town. Supporting documents and other secondary data were also gathered through this exercise. The study is currently at an advanced stage of data analysis and the initial written report is expected during the first half of 2016.

## Discussion

Our work will directly strengthen health systems and inform educational programs of the requirements to meet minimum standards. Our program of research will inform policy makers and health care managers in planning and implementing effective health intervention programs that successfully reach all targeted populations, particularly the most vulnerable. This program of research will inform the design of strategies to control and treat hypertension. This will help prevent complications and more importantly reduce the risks of cardiovascular and cerebrovascular diseases, as it is well established that hypertension is a key contributor to their development. As such, our program of research will contribute to the secondary prevention of worsening hypertension and of comorbidities associated with hypertension. Our findings could aid the development of practical guides for delivering long-term medical care for hypertension within weak health care systems characteristic of resource-limited settings such as SSA. Many African countries look towards SSA for infrastructure and guidance when addressing their health system’s needs. In addition, policies and recommendations from international organizations could be informed by our research, which will therefore assist with better targeted funding. The possibility of applying our findings to other countries or regions adds great value to our work.

## Appendix

Multimedia Appendix 1 IDRC peer-review report.

Multimedia Appendix 2 Confirmation of Defense Thesis Proposal.

Multimedia Appendix 3 Full Ethics Approval University of Ottawa.

Multimedia Appendix 4 Ethics Approval South Africa Medical Research Council.

## Abbreviations

DOH: Department of Health
GACD: Global Alliance for Chronic Disease
MRC: Medical Research Council
NCD: noncommunicable diseases
SAHANES: South African National Health and Nutrition Examination Survey
TB: Tuberculosis
UN: United Nations
WHO: World Health Organization
