# Supplementary material for: Health Systems Readiness to Manage the Hypertension Epidemic in Primary Health Care Facilities in the Western Cape, South Africa: A Study Protocol
Source: JMIR Res Protoc. 2016 Feb 29;5(1):e35. doi: 10.2196/resprot.5381 (PMC4791525; doi:10.2196/resprot.5381)
Supplement: Multimedia Appendix 1 [file resprot_v5i1e35_app1.pdf]

Rodrigue DEUBOUE <rdeuboue@gmail.com>

Results of IDRC Doctoral Research Award Competition of April 2014

9 messages

Liliane Castets-Poupart <lcastets-poupart@idrc.ca> Wed, Aug 13, 2014 at 5:00 PM
To: "rdeuboue@gmail.com" <rdeuboue@gmail.com>

Attention: Rodrigue Innocent Deuboue Tchialeu

Dear Mr. Deuboue Tchialeu:

It is a great pleasure to inform you that you have been recommended for an IDRC Doctoral Research Award **on a conditional basis**.

The award will cover expenses related to your field research as of **August 1, 2014.**

1. Review of your file

Your application was recommended for an award on the condition that you include and respond to the comments made by the evaluators.

Please find attached some of the comments made by the evaluators. Please address these comments and explain how you will incorporate them into your research. You are not required to submit a revised research proposal. Your reply will be subject to further review prior to granting the award.

2. Budget

We will review the budget that you have submitted to determine which expenses are acceptable and confirm the actual amount to be granted to you.

3. Contract

You have up to 12 months from the above-cited date to start your field research. The items listed below are necessary for us to process your award contract. Please send the information **if it has not been sent already.** You can send one document at a time or all at the same time.

a. An updated budget when specifically requested in the comments.

b. An updated schedule of the field research to be funded.

c. Proof of affiliation (scanned copy of an original letter) with an institution in the country or region in which you will do your research, **if not already provided**. Please note that obtaining visas or special country clearance for undertaking research is the responsibility of the award holder.

d. Confirmation by email from your supervisor that all comprehensive exams and courses have been passed.

e. Reply to the comments made by the evaluators.

There is no urgency to reply to this message. However, any delay in responding could delay your departure to the field. Please note that before we can issue the contract and release the funds, we need to review and approve the above documents. It may take up to a month after the review and approval to prepare the contract, have it signed and transfer the funds to your account. For this reason, we recommend that you do not wait until the last minute before your trip to respond to this message. Please direct your reply and the requested documents to my colleague, Mr. Jean-Claude Dumais, Awards Officer, E-Mail: jdumais@idrc.ca, (tel.: 613-236-6163 ext. 2430 or 613-696-2430).

On behalf of IDRC, I offer my congratulations and wish you success in your research.

Yours sincerely,

Liliane Castets-Poupart
Program Management Officer | Agente de gestion de programme
Fellowships and Awards | Programme de bourses
International Development Research Centre | Centre de recherches pour le développement

Evaluators' comments for Rodrigue Deuboue Tchialeu IDRA_April 2014 competition

Comments from the Evaluators for: Rodrigue Deuboue Tchialeu

Evaluator # 1:

This is clearly an experienced, skilled and highly motivated candidate who has background and interest in undertaking relevant delivery modelling operational research. The results could be relevant for anti-hypertension treatment strategies, particularly in the Western Cape. Given that the research will complement additional research being supported at the MRC in South Africa, there is every chance that the results will be used.

The overall research questions are clearly indicated. The objective flows from the questions but it is extremely weak when the justification for choosing South Africa is discussed.

The challenge is that the candidate is applying a very technical approach, in a siloed manner, to a very complex and challenging context. There may be challenges in fully understanding the dynamics of procurement and delivery; there may be challenges in understanding the needs of the vulnerable members of the population (who don't even present to health facilities for treatment). And there ultimately could be policy development and implementation challenges given that the proposed needs are not being costed. This could all result in a less than fully accurate picture being created and that the results might not be usable. It is doubtful that the 'good mix' identified for Western Cape South Africa will be attainable for many parts of Sub-Saharan Africa and thus the opportunities for being a reference point are somewhat limited.

It is therefore important to carefully frame the proposed research so that its reach and use is realistic and related to the specific context where it might be relevant. It is important to prepare for and build in approaches to address the 'soft issues' of conducting the research in a highly politicized environment as an outsider. And it is important to ensure that the needs of the vulnerable members of the society are identified and fed into any model to ensure that a relevant picture is developed.

Evaluator # 2:

Strengths:

1. The applicant has achieved well academically and has assembled a good proposal.
2. Engagement and support from the MRC-SA is a great bonus.
3. The applicant has made a strong effort to address the weaknesses raised previously by the reviewers.
4. It was great to see mention of power and agency issues in the section on gender. I hope this perspective on gender dynamics will be operationalized and adhered to during the course of the project.

Weaknesses:

1. Given that there will be six public health centres as part of the project, I hope that contextual factors such as (their size, their capacity to provide services, proximity to roads and suppliers, etc.) would be incorporated into the analysis.
2. The proposed activities under 'Dissemination' are very broad and a bit generic. Beyond the papers that will be produced, putting more thought into how social media, news outlets and other channels can be strategically used should be considered from the outset of the project.
3. Some skills in complex adaptive systems would be helpful to add.
4. The section on ethics can be strengthened. There is nothing blatantly missing or off-putting. However, more emphasis on beneficence and non-maleficence would strengthen the approach and further ensure the research participants (or the patients they serve) are not harmed in any way.

All these points must be addressed as a condition of the award.

IDRC

Responses Comments from the Evaluators for: RodrigueDeubouеTchialeu

Evaluator # 1:

This is clearly an experienced, skilled and highly motivated candidate who has background and interest in undertaking relevant delivery modelling operational research. The results could be relevant for anti-hypertension treatment strategies,particularly in the Western Cape. Given that the research will complement additional research being supported at the MRC in South Africa, there is every chance that the results will be used.

The overall research questions are clearly indicated. The objective flows from the questions but it is extremely weak when the justification for choosing South Africa is discussed.

Our response: Thank you for your comments. The issue of managing the hypertension epidemic that we are planning to address with our research is common with Sub-Saharan Africa countries with weak health systems. This means that there were many other countries where we could have elect to conduct our research but the choice of South Africa was mostly motivated by the presence of the MRC which has conducted substantial research on issues of NCDs. As this study is part of my PhD, it was important to find an environment where we could get adequate supervision and support. South Africa through the affiliation with the MRC offered us better conditions to conduct the research and the choice was validated by my supervisors and the University of Ottawa.

The challenge is that the candidate is applying a very technical approach, in a siloed manner, to a very complex and challenging context. There may be challenges in fully understanding the dynamics of procurement and delivery; there maybe challenges in understanding the needs of the vulnerable members of the population (who don't even present to healthfacilities for treatment). And there ultimately could be policy development and implementation challenges given that theproposed needs are not being costed. This could all result in a less than fully accurate picture being created and that theresults might not be usable. It is doubtful that the 'good mix' identified for Western Cape South Africa will be attainable formany parts of Sub-Saharan Africa and thus the opportunities for being a reference point are somewhat limited.

Our response: Thank you for these pertinent remarks. The challenges described here are real and it is the reason why we intend to spend a year on the ground not only to interview key informants and stakeholders but also to go back to them in order to validate the picture that we will build based on the data collected after analysis. It will be an iterative process that will allow us to get as close as possible to the real picture of the procurement and delivery systems. We believe that this participative approach will also help to create favourable conditions for policy development and implementation. The outcome of this research will be translate in concrete recommendations on how to go

about managing the hypertension epidemic and further research could be initiated to study the costing of implementing these recommendations.
It is true that just having good practices implemented in Western Cape will not make it automatically a reference point for Sub-Saharan Africa. There is considerable work that needs to be done on the dissemination aspect in order to see that happen. We do not intend to stop with this PhD research project. We plan to expand this research and make its results usable beyond Western Cape and South Africa but some of these are beyond the scope of this initial research project. We are fully aware of the potential to expand this research later on.

It is therefore important to carefully frame the proposed research so that its reach and use is realistic and related to the specific context where it might be relevant. It is important to prepare for and build in approaches to address the 'softissues' of conducting the research in a highly politicized environment as an outsider. And it is important to ensure that the needs of the vulnerable members of the society are identified and fed into any model to ensure that a relevant picture is developed.

Our response: These remarks are very welcome and we will definitely put them in practice as we move forward with our project. We have been experiencing those ‘softissues’ already as we go through the ethics approval in South Africa. The propose timeline for our research also takes this label of ‘outsider’ in consideration and that is another reason why we plan to stay in the field for a good 12 months period.

Evaluator # 2:

Strengths:

1. The applicant has achieved well academically and has assembled a good proposal.
2. Engagement and support from the MRC-SA is a great bonus.
3. The applicant has made a strong effort to address the weaknesses raised previously by the reviewers.
4. It was great to see mention of power and agency issues in the section on gender. I hope this perspective on genderdynamics will be operationalized and adhered to during the course of the project.

Our response: Thank you for your appreciation. No further comments.

Weaknesses:
1. Given that there will be six public health centres as part of the project, I hope that contextual factors such as (their size,their capacity to provide services, proximity to roads and suppliers, etc.) would be incorporated into the analysis.

Our response: Thank you for your suggestion. Yes those contextual factors will be accounted for in the analysis.

2. The proposed activities under 'Dissemination' are very broad and a bit generic. Beyond the papers that will beproduced, putting more thought into how social media, news outlets and other channels can be strategically used should be considered from the outset of the project.

Our response: Thank you for your suggestion that we will definitely take in consideration to improve our dissemination strategy. We intend to adapt our dissemination strategies to our target audience characteristics. At the local level, we intend to investigate the most effective channels that will allow us to disseminate our results and this could be very much contextualized to the local environment. We have considered online blogs, professional forums, interventions on news programs but these strategies will be finalized only once we are on the field experiencing local realities.
Our approach to dissemination is that it will be an ongoing process and spending 12 months on the ground will allow us to make sure that research participants are fed back with the results of our research.

3. Some skills in complex adaptive systems would be helpful to add.

Our response: Thank you for your suggestion. Indeed we are working on furthering our knowledge on that and we have also secured additional help from the University of Ottawa to assist us in that aspect of the research project. Our supervisors understood the need to bring on board someone with expertise in complex adaptive systems and modelling to assist us on this research project.

4. The section on ethics can be strengthened. There is nothing blatantly missing or off-putting. However, more emphasis on beneficence and non-maleficence would strengthen the approach and further ensure the research participants (or thepatients they serve) are not harmed in any way.

Our response: Thank you for your suggestion. We will include a paragraph on beneficence and non-maleficence in the ethics session.

All these points must be addressed as a condition of the award.
