# Supplementary material for: Health Systems Readiness to Manage the Hypertension Epidemic in Primary Health Care Facilities in the Western Cape, South Africa: A Study Protocol
Source: JMIR Res Protoc. 2016 Feb 29;5(1):e35. doi: 10.2196/resprot.5381 (PMC4791525; doi:10.2196/resprot.5381)
Supplement: Multimedia Appendix 2 [file resprot_v5i1e35_app2.pdf]

uOttawa

Université d'Ottawa
Faculté des études supérieures et postdoctorales
Programme de doctorat en santé des populations

University of Ottawa
Faculty of Graduate and Postdoctral Studies
Doctoral Program in Population Health

POP9997
Soutenance de projet de thèse / Thesis Proposal Defense

Étudiant<br>Student	Rodrigue Innocent Deuboué Tchialeu (6988101)
Titre du projet<br>Project title	Health Systems Readiness to Control the Hypertension Epidemic in Developing Countries : Investigations in South Africa
Date	February 26, 2014

		Approuvé (révisions suggérées) L'étude peut débuter, sujet à l'approbation déontologique. Note finale « satisfaisant »	Approuvé en principe (révisions requises) L'étude peut débuter sujet à l'approbation déontologique. Note finale « satisfaisant »	Non satisfaisant (révisions requises, resoumettre et soutenir à nouveau) Indiquer les raisons sur une feuille séparée attachée à ce formulaire. Note finale « non satisfaisant »
		Approved (suggested revisions) The study can begin subject to ethics approval. Final grade "satisfactory"	Approved in principle (revisions required) The study can begin subject to Ethics approval. Final grade "satisfactory"	Not satisfactory (revisions required, resubmit and defend again) Indicate the reasons on a separate sheet appended to this form. Final grade "not satisfactory"
I	Introduction, cadre théorique et revenue de la littérature<br>Introduction, theoretical framework and literature review	✓		
II	Objectifs et hypotheses<br>Objectives and hypotheses	✓		
III	Méthodologie<br>Methodology	✓		
IV	Analyses<br>Analysis	✓		
V	Document global<br>Overall document	✓		

Sanni Yaya (Thesis supervisor) [signature]

Ronald Labonté (Thesis co-supervisor) ____________

Rodrigue Innocent Deuboué Tchialeu (Student) [signature]

Ivy Bourgeault (Member) [signature]

André Pascal Kengne (Member) Video conference

Douglas E. Angus (Extra Examiner) Douglas E. Angus

Louise Bouchard (Chair and Program Director) Louise Bouchard

Date: 26 février 2014

613-562-5691
613-562-5112
1 Stewart (300)
Ottawa ON K1N 6N5 Canada
www.uOttawa.ca
