# Supplementary material for: Health Systems Readiness to Manage the Hypertension Epidemic in Primary Health Care Facilities in the Western Cape, South Africa: A Study Protocol
Source: JMIR Res Protoc. 2016 Feb 29;5(1):e35. doi: 10.2196/resprot.5381 (PMC4791525; doi:10.2196/resprot.5381)
Supplement: Multimedia Appendix 3 [file resprot_v5i1e35_app3.pdf]

Université d'Ottawa
Bureau d'éthique et d'intégrité de la recherche

University of Ottawa
Office of Research Ethics and Integrity

Ethics Approval Notice

Health Sciences and Science REB

Principal Investigator / Supervisor / Co-investigator(s) / Student(s)

First Name	Last Name	Affiliation	Role
Sanni	Yaya	Health Sciences / Others	Supervisor
Rodrigue Innocent	Deuboue Tchialeu	Health Sciences / Population Health	Student Researcher

File Number: H03-14-18

Type of Project: PhD Thesis

Title: Health Systems Readiness to Control the Hypertension Epidemic in Developing Countries : Investigations in South Africa

Approval Date (mm/dd/yyyy)	Expiry Date (mm/dd/yyyy)	Approval Type
10/16/2014	10/15/2015	Ia

(Ia: Approval, Ib: Approval for initial stage only)

Special Conditions / Comments:
N/A

550, rue Cumberland, pièce 154
Ottawa (Ontario) K1N 6N5 Canada
550 Cumberland Street, room 154
Ottawa, Ontario K1N 6N5 Canada
(613) 562-5387 • Téléc./Fax (613) 562-5338
www.recherche.uottawa.ca/deontologie/ www.research.uottawa.ca/ethics/

Université d'Ottawa
Bureau d'éthique et d'intégrité de la recherche

University of Ottawa
Office of Research Ethics and Integrity

This is to confirm that the University of Ottawa Research Ethics Board identified above, which operates in accordance with the Tri-Council Policy Statement (2010) and other applicable laws and regulations in Ontario, has examined and approved the ethics application for the above named research project. Ethics approval is valid for the period indicated above and subject to the conditions listed in the section entitled "Special Conditions / Comments".

During the course of the project, the protocol may not be modified without prior written approval from the REB except when necessary to remove participants from immediate endangerment or when the modification(s) pertain to only administrative or logistical components of the project (e.g., change of telephone number). Investigators must also promptly alert the REB of any changes which increase the risk to participant(s), any changes which considerably affect the conduct of the project, all unanticipated and harmful events that occur, and new information that may negatively affect the conduct of the project and safety of the participant(s). Modifications to the project, including consent and recruitment documentation, should be submitted to the Ethics Office for approval using the "Modification to research project" form available at: http://www.research.uottawa.ca/ethics/forms.html.

Please submit an annual report to the Ethics Office four weeks before the above-referenced expiry date to request a renewal of this ethics approval. To close the file, a final report must be submitted. These documents can be found at: http://www.research.uottawa.ca/ethics/forms.html.

If you have any questions, please do not hesitate to contact the Ethics Office at extension 5387 or by e-mail at: ethics@uOttawa.ca.

Signature:

Riana Marcotte

Riana Marcotte
Protocol Officer for Ethics in Research
For Daniel Lagarec, Chair of the Health Sciences and Sciences REB

550, rue Cumberland, pièce 154
Ottawa (Ontario) K1N 6N5 Canada

550 Cumberland Street, room 154
Ottawa, Ontario K1N 6N5 Canada

(613) 562-5387 • Téléc./Fax (613) 562-5338
www.recherche.uottawa.ca/deontologie/ www.research.uottawa.ca/ethics/
