# Supplementary material for: Health Systems Readiness to Manage the Hypertension Epidemic in Primary Health Care Facilities in the Western Cape, South Africa: A Study Protocol
Source: JMIR Res Protoc. 2016 Feb 29;5(1):e35. doi: 10.2196/resprot.5381 (PMC4791525; doi:10.2196/resprot.5381)
Supplement: Multimedia Appendix 4 [file resprot_v5i1e35_app4.pdf]

SOUTH AFRICAN MEDICAL RESEARCH COUNCIL
MRC SOUTH AFRICA

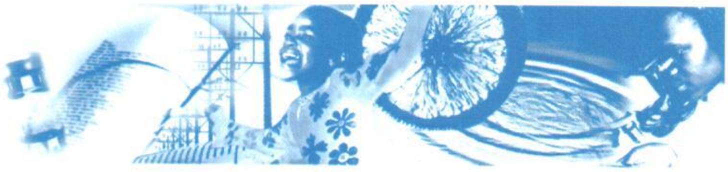

ETHICS COMMITTEE

PO Box 19070, 7505 Tygerberg, South Africa
Francie van Zijl Drive, Parowvallei, 7500
Tel: +27 (0)21 938-0687; Fax: +27 (0) 866-854023
E-mail: adri.labuschagne@mrc.ac.za
http://www.mrc.ac.za/ethics/ethics.htm

15 October 2014

To whom it may concern

Protocol ID: EC011-7/2014
Protocol title: Health systems readiness to control the hypertension epidemic in developing countries: investigations in South Africa

I would like to confirm that the above-mentioned research protocol submitted by Mr Rodrigue Deuboué, who will be working in the Non-Communicable Diseases Research Unit, has successfully undergone a scientific review process. Two reviewers have reviewed it and have confirmed that her comments and concerns were addressed satisfactorily.

Yours sincerely

A. Labuschagne

Ms A Labuschagne
Ethics Officer
